# *Echinococcus granulosus* ubiquitin-conjugating enzymes (E2D2 and E2N) promote the formation of liver fibrosis in TGFβ1-induced LX-2 cells

**DOI:** 10.1186/s13071-024-06222-8

**Published:** 2024-04-20

**Authors:** Xiaodi Du, Ruiqi Hua, Xue He, Wei Hou, Shengqiong Li, Aiguo Yang, Guangyou Yang

**Affiliations:** 1 Department of Parasitology, College of Veterinary Medicine, Sichuan Agricultural University, Chengdu, 611130 China; 2 Sichuan Center for Animal Disease Control and Prevention, Chengdu, 610041 China

**Keywords:** *Echinococcus granulosus*, E2D2, E2N, Liver fibrosis, LX-2 cells

## Abstract

**Background:**

Cystic echinococcosis (CE) is a widespread zoonosis caused by the infection with *Echinococcus granulosus* sensu lato (*E. granulosus* s.l.). CE cysts mainly develop in the liver of intermediate hosts, characterized by the fibrotic tissue that separates host organ from parasite. However, precise mechanism underlying the formation of fibrotic tissue in CE remains unclear.

**Methods:**

To investigate the potential impact of ubiquitin-conjugating enzymes on liver fibrosis formation in CE, two members of ubiquitin-conjugating (UBC) enzyme of *Echinococcus granulosus* (*Eg*E2D2 and *Eg*E2N) were recombinantly expressed in *Escherichia coli* and analyzed for bioinformatics, immunogenicity, localization, and enzyme activity. In addition, the secretory pathway and their effects on the formation of liver fibrosis were also explored.

**Results:**

Both r*Eg*E2D2 and r*Eg*E2N possess intact UBC domains and active sites, exhibiting classical ubiquitin binding activity and strong immunoreactivity. Additionally, *Eg*E2D2 and *Eg*E2N were widely distributed in protoscoleces and germinal layer, with differences observed in their distribution in 25-day strobilated worms. Further, these two enzymes were secreted to the hydatid fluid and CE-infected sheep liver tissues via a non-classical secretory pathway. Notably, TGFβ1-induced LX-2 cells exposed to r*Eg*E2D2 and r*Eg*E2N resulted in increasing expression of fibrosis-related genes, enhancing cell proliferation, and facilitating cell migration.

**Conclusions:**

Our findings suggest that *Eg*E2D2 and *Eg*E2N could secrete into the liver and may interact with hepatic stellate cells, thereby promoting the formation of liver fibrosis.

**Graphical Abstract:**

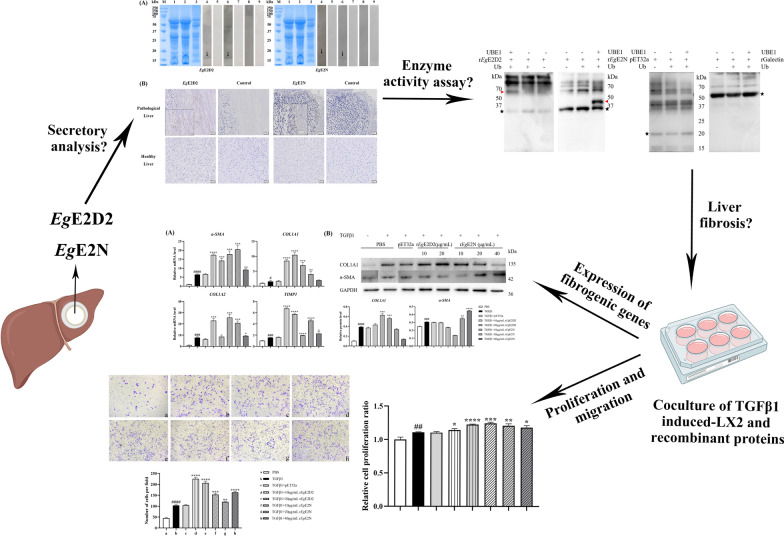

**Supplementary Information:**

The online version contains supplementary material available at 10.1186/s13071-024-06222-8.

## Background

Cystic echinococcosis (CE) is a severe zoonotic parasitic disease caused by the metacestode of *Echinococcus granulosus* sensu lato (*E. granulosus* s.l.). It affects wild mammalians, domestic livestock, and humans, posing a significant threat to both human beings and animal husbandry. CE is distributed around most of the world, with high prevalence observed in central Asia, eastern Africa, and Mediterranean countries [1]. Neglecting the presence of CE could lead to a considerable increase in mortality rates, although the mortality rate is relatively low, ranging from 2% to 4% [2]. The World Organization for Animal Health (OIE) has classified CE as the third largest food-borne parasitic disease worldwide [3]. Furthermore, CE is included among the 17 neglected diseases identified by the World Health Organization (WHO) as targets for control in hyperendemic countries by 2030 [4].

Parasitism of *E*. *granulosus* s.l. is primarily characterized by the growth of cysts in the liver and lung of the intermediate hosts [5]. The cyst structure consists of three layers including the germinal layer, laminated layer, and adventitial layer. Specifically, the adventitial layer is formed as a fibrotic tissue to isolate and limit the development of the parasitic cysts. In terms of parasites, it acts as a protective shell that separates the germinal layer from surrounding infiltrating immune cells [6]. In addition, it may also limit the efficacy of drugs targeting the parasite [7]. Research on the mechanism of liver fibrosis caused by infections from parasites is currently lacking. Therefore, elucidating the mechanism of fibrotic tissue caused by *E*. *granulosus* s.l. holds potential clinical significance not only for the treatment of CE, but for other parasitic diseases as well.

Ubiquitin-conjugating enzyme (E2) is one of the three key enzymes needed for ubiquitination of proteins. In eukaryotic genomes, there are 16–35 members of the E2 family [8]. Most E2s possess a highly conserved ubiquitin-conjugating domain known as the UBC domain, which facilitates the activation of ubiquitin/ubiquitin-like (Ub/Ubl) proteins in the presence of adenosine triphosphate (ATP) [9]. Ubiquitin is continuously transferred to the target protein, leading to the formation of mono-ubiquitination, multi-mono-ubiquitination, or poly-ubiquitination [10]. Mono-ubiquitination regulates various processes such as endocytosis, protein localization, and transport [11]. E2s determine the common Ub linkages in poly-ubiquitination, including Lys11, Lys48, and Lys63. Specifically, Lys11 and Lys48 chains are usually involved in proteasome degradation, while Lys63 chain participates in non-proteolytic processes, such as immune regulation, DNA repair, and signal transduction [12].

Currently, functional studies on parasite E2s are limited to *Entamoeba histolytica* [13], *Trypanosoma brucei* [14], and *Trichinella spiralis* [15]. For instance, the secretory proteins of *Trichinella spiralis* contains E2 Ub-conjugating activity, which is attributed to *Ts*UBE2L3. Expression of *Ts*UBE2L3 in mouse skeletal muscle cells leads to the down-regulation of protein ubiquitination, primarily affecting movement, sarcoma, and extracellular matrix (ECM) proteins, highlighting the significance of E2s in parasite–host interactions [15]. In this study, we screened *Eg*E2D2 and *Eg*E2N from *E. granulosus* proteomic data [16, 17]. The recombinant proteins were utilized for Western blotting, immunofluorescence localization, and secretory analysis, and their effects on the expression of fibrogenic genes, cell proliferation, and migration in TGFβ1-induced LX-2 were also investigated. Our findings provide a foundation for underlying the formation of fibrotic tissue in CE.

## Methods

### Animals and cell line

Nine female SD rats (4 weeks old) were purchased from Dashuo Experimental Animal (Chengdu, China; experimental animal production license no. SCXK 2020–030). They were provided with food pellets and sterilized water ad libitum. The immortalized human hepatic stellate cell line (LX-2, MeisenCTCC, China) was cultured in DMEM (Gibco, USA) supplemented with 10% (vol/vol) FBS (NEWZERUM, New Zealand) and 100 U/mL penicillin–streptomycin. The LX-2 cells were cultured at 37 °C in a humid atmosphere of 5% CO_2_, and the medium was changed every day.

### Parasites, sera, tissue, and small extracellular vesicles

Hydatid cysts, hepatic tissue, protoscoleces (PSCs), and 25-day strobilated worms were provided by the Department of Parasitology, College of Veterinary Medicine, Sichuan Agricultural University. Healthy sheep livers and negative sera were obtained from sheep free of tapeworm infection. Following the method described in a previous study [17], fertile and sterile cysts were distinguished, meanwhile, hydatid fluid (HF) and germinal layer were aseptically isolated. The morphological characteristics of the samples were observed by hematoxylin–eosin staining (HE) (Additional file 1: Fig. S1). Small extracellular vesicles (sEV) originating from HF were isolated and identified according to previously described methods [18, 19]. The morphology and size of sEV were determined using transmission electron microscope and nanoparticle tracking analysis (Additional file 2: Fig. S2).

### Bioinformatic analysis

For cloning and sequencing, primers were designed on the basis of the sequences in the GenBank database (*EgE2D2*: MK534430.1; *EgE2N*: XM_024494499.1). The sequenced *Eg*E2D2 and *Eg*E2N were further subjected to open reading frames (ORFs) prediction (https://www.ncbi.nlm.nih.gov/orffinder/), and basic physicochemical properties were predicted using the Expasy website (https://web.expasy.org/protparam/). Prediction of signal peptides and transmembrane regions was performed using SignalP 5.0 (http://www.cbs.dtu.dk/Services/SignalP/) and TMHMM 2.0 (http://www.cbs.dtu.dk/services/TMHMM-2.0), respectively. Subcellular localization and active site prediction were predicted using Cell-PLoc (http://www.csbio.sjtu.edu.cn/bioinf/Cell-PLoc-2/) and InterPro (http://www.ebi.ac.uk/interpro/), respectively. NPS (https://npsa-prabi.ibcp.fr/cgi-bin/npsa_automat.pl?page=/NPSA/npsa_sopma.html) and SWISS-MODEL (https://swissmodel.expasy.org/interactive) were utilized for secondary and tertiary structure predictions, respectively.

### Production and purification of recombinant EgE2D2 and rEgE2N

Total RNA was extracted from PSCs using the RNAprep pure Tissue Kit (Tiangen, China) according to the manufacturer’s guidelines, and the Thermo Scientific RevertAid (Thermo Fisher Scientific, USA) was used to synthesize first-strand cDNA. The sequences of *EgE2D2* and *EgE2N* were amplified with primers described in Table 1. Purified polymerase chain reaction (PCR) products were cloned into a pET32a (+) vector and transformed into *E. coli* BL21 (DE3). The BL21 strain was cultured at 37 °C, 160 rpm for 6 h, and then added with 1 mM isopropyl β-D-1-thiogalactopyranoside (IPTG) to induce protein expression. Ni^2+^ affinity chromatography column (Bio-Rad, USA) was used to purify the recombinant proteins.

**Table 1:** Primer sequences

Gene	Forward primer (5′-3′)	Reverse primer (5′-3′)
EgE2D2	C***GGATCC***ATGGCCCTGAAGAGGATTC	CG***GAATTC***CTACATTGCGTACTTCTGAGTCC
EgE2N	C***GAGCTC***ATGAGTGGACATCTTCCTACAC	C***CTCGAG***CTAGCGGAAGTCGGAAG
α-SMA	GACAGCTACGTGGGTGACGAA	TTTTCCATGTCGTCCCAGTTG
COL1A1	GTGCGATGACGTGATCTGTGA	CGGTGGTTTCTTGGTCGGT
COL1A2	GTTGCTGCTTGCAGTAACCTT	AGGGCCAAGTCCAACTCCTT
TIMP1	TGCAGGATGGACTCTTGCAC	GCATTCCTCACAGCCAACAG
GAPDH	CAAGGTCATCCATGACAACTTTG	GTCCACCACCCTGTTGCTGTAG

Black and bold italics signify restriction enzyme sites

### Preparation of polyclonal antibody

Polyclonal antibodies against recombinant *Eg*E2D2 (r*Eg*E2D2) and *Eg*E2N (r*Eg*E2N) were prepared as previously described [20]. Briefly, rats were subcutaneously injected four times (at an interval of 1 week). The first immune reagent was 200 μg recombinant protein emulsified with equal volume of Freund’s complete adjuvant (Sigma, USA), and the booster reagent was 200 μg recombinant protein emulsified with equal volume of Freund’s incomplete adjuvant. Blood samples were collected to isolate antiserum, and the serum titer was determined by ELISA. Purification of immunoglobulin G (IgG) was performed using Protein G Resin FF Prepacked Column (GenScript, China), and purification efficiency was assessed by Sodium Dodecyl Sulphate-Polyacrylamide Gel Electrophoresis (SDS-PAGE) analysis.

### Western blotting analysis

The animal total protein extraction kit (Bestbio, China) and RIPA lysis buffer (Solarbio, China) were used for protein extraction of PSCs and LX-2 cells, respectively. After quantifying protein concentration using BCA kit (Bestbio, China), 20 μg of protein samples were separated in 12% SDS-PAGE gel and transferred to nitrocellulose membranes (Merck, Germany). The membrane was sealed with 5% skimmed milk at room temperature for 2 h, and then the primary antibodies, CE positive/negative sheep sera, polyclonal antibodies (anti-r*Eg*E2D2 and r*Eg*E2N), pre-immunized rat sera (1:200 *v*/*v* dilution), α-SMA, COL1A1, and GAPDH (1:5000 *v*/*v* dilution, ABclonal, China) were incubated overnight at 4 °C. After fully washing the membrane, corresponding horseradish peroxidase (HRP)-labeled secondary antibodies (goat anti-rat IgG, goat anti-rabbit IgG, and rabbit anti-sheep IgG (1:2000 *v*/*v* dilution, ABclonal, China) were added and incubated at room temperature for 1 h. Metal Enhanced DAB Substrate Kit (20 ×) (Solarbio, China) was used to visualize the bands.

### Immunohistochemistry

Immunohistochemistry was performed following a previous protocol [20]. Slices of PSCs, 25-day strobilated worms, and fertile and sterile cyst walls were incubated overnight at 4 °C with anti-r*Eg*E2D2/anti-r*Eg*E2N rat IgG or pre-immunized rat serum (1:200 *v*/*v* dilution). Subsequently, the slices were incubated with fluorescein isothiocyanate (FITC)-conjugated goat anti-rat IgG (1:2000 dilution, Thermo Fisher Scientific, USA) at room temperature in the dark for 1 h. Nuclei were stained with DAPI (1 mg/mL, Solarbio, China) for 7 min. After each incubation, slices were washed with Phosphate Buffered Saline (PBS) for 5 min × four times. Finally, the slices were sealed with anti-fluorescence quenching agent and observed under Olympus fluorescence microscope (Olympus, Japan).

After the samples (infected and healthy liver tissue) were fixed, embedded, and sliced, and antigen retrieval was performed, 3% H_2_O_2_ was added and incubated at room temperature for 20 min. The primary antibody was incubated as described above, and the secondary antibody (a goat anti-rat IgG labeled with HRP, 1:2000 *v*/*v* dilution, ABclonal, China) was incubated at room temperature for 30 min. Fresh chromogenic liquid droplet prepared with Metal Enhanced DAB Substrate Kit (20×) was added to the tissue, and the brownish-yellow or brown color was observed under the microscope as a positive signal. The slices were immersed in hematoxylin dye solution for 3 min, then dehydrated and sealed.

### Enzyme activity assay

To determine whether r*Eg*E2D2 and r*Eg*E2N have Ub conjugating activity, pET32a protein and Galectin were used as controls for ubiquitination in vitro. The addition amount of each component in the reaction system was carried out according to the previous description [21]. Non-reductive [Dithiothreitol (DTT) free] SDS loading buffer was added to the reaction solution and boiled for 5 min. Western blotting analysis was performed after 12% SDS-PAGE gel electrophoresis. The mouse anti-His tag mAb was used as primary antibody. The Ub conjugating activity of E2 was determined by the molecular weight of Ub.

### Cell viability assay

Endotoxin Removal Kit (Smart-Lifesciences, China) was used to remove endotoxin from the recombinant proteins, and the concentration of endotoxin was determined according to ToxinSensor^™^ Chromogenic LAL Endotoxin Assay Kit (GenScript, China) to ensure that endotoxin level was below 0.02 EU/mL. Potential cytotoxic effects of recombinant proteins on LX-2 cells were evaluated by assessing cell viability using the Cell Counting Kit-8 (CCK-8, Beyotime, China) as described previously [22]. Briefly, 100 μL cell suspension (2 × 10^4^ cells) was added to the 96-well plate. After 24 h, different concentrations of recombinant protein (0, 10, 20, 40, 60, 80 μg/mL) were added and incubated at 37 °C for another 24 h. Subsequently, the culture medium was replaced with fresh medium, CCK-8 working solution was added and incubated at 37 °C for 1 h, and the absorbance was measured at 450 nm.

### Cell activation assay

A total of 2 × 10^5^ cells were added to the 6-well plate. Once the cells reached 80% confluence, they were incubated with or without TGFβ1 (5 ng/mL) for 12 h, and then cultured with different concentrations of recombinant protein for 24 h [23]. The expression levels of fibrogenic genes (*α-SMA*, *COL1A1*, *COL1A2*, and *TIMP1*) were detected by quantitative reverse transcription (qRT)-PCR using the primers presented in Table 1. The qRT-PCR reaction mixture consisted of 12.5 μL TB Green^™^ Premix Ex Taq^™^ II (TaKaRa, Japan), 1 μL each primer, 2 μL cDNA, and 8.5 μL ddH_2_O. Samples were heated for 30 s at 95 °C and a two-step cycle (5 s at 95 °C, 60 s at 60 °C) was repeated for 40 cycles. The GAPDH (GenBank: DQ403057) gene was selected as the housekeeping gene, and the relative expression levels of fibrogenic genes were calculated using 2^−△△Ct^ method [24]. The protein expression levels of α-SMA and COL1A1 were detected by Western blotting. Image Lab 5.0 was used to determine the gray value, and GAPDH was employed as a loading control.

### Cell proliferation and migration assays

Cell proliferation was evaluated by CCK-8 assay [25]. LX-2 cells were cultured in 96-well plate, incubated with 5 ng/mL TGFβ1 for 12 h, and then cultured with different concentrations of recombinant protein for 24 h. After replacing the culture medium, the absorbance was measured by adding CCK-8 working solution and incubating at 37 °C for 1 h at 450 nm.

Cell migration was evaluated using a 24-well transwell plate with 8.0 μm pore polycarbonate membrane insert, and 5 ng/mL TGFβ1 pre-stimulated cells were harvested and resuspended in the medium. A total of 3 × 10^4^ cells was loaded into the upper chamber, while the lower chamber filled 600 μL medium containing different concentrations of recombinant protein dissolved in PBS. The chamber was further incubated for 24 h at 37 °C. The staining and counting of the cells in five random regions were performed under optical microscope.

### Statistics analysis

All data were presented as means ± standard deviation (SD) from multiple experiments. Statistics analysis was performed with Student’s *t* test method. *P*-values less than 0.05 were defined as the threshold for statistical significance. The significance levels were denoted as follows: *, *P* < 0.05; **, *P* < 0.01; ***, *P* < 0.001.

## Results

### Sequence analyses of EgE2D2 and EgE2N

**Fig. 1:**
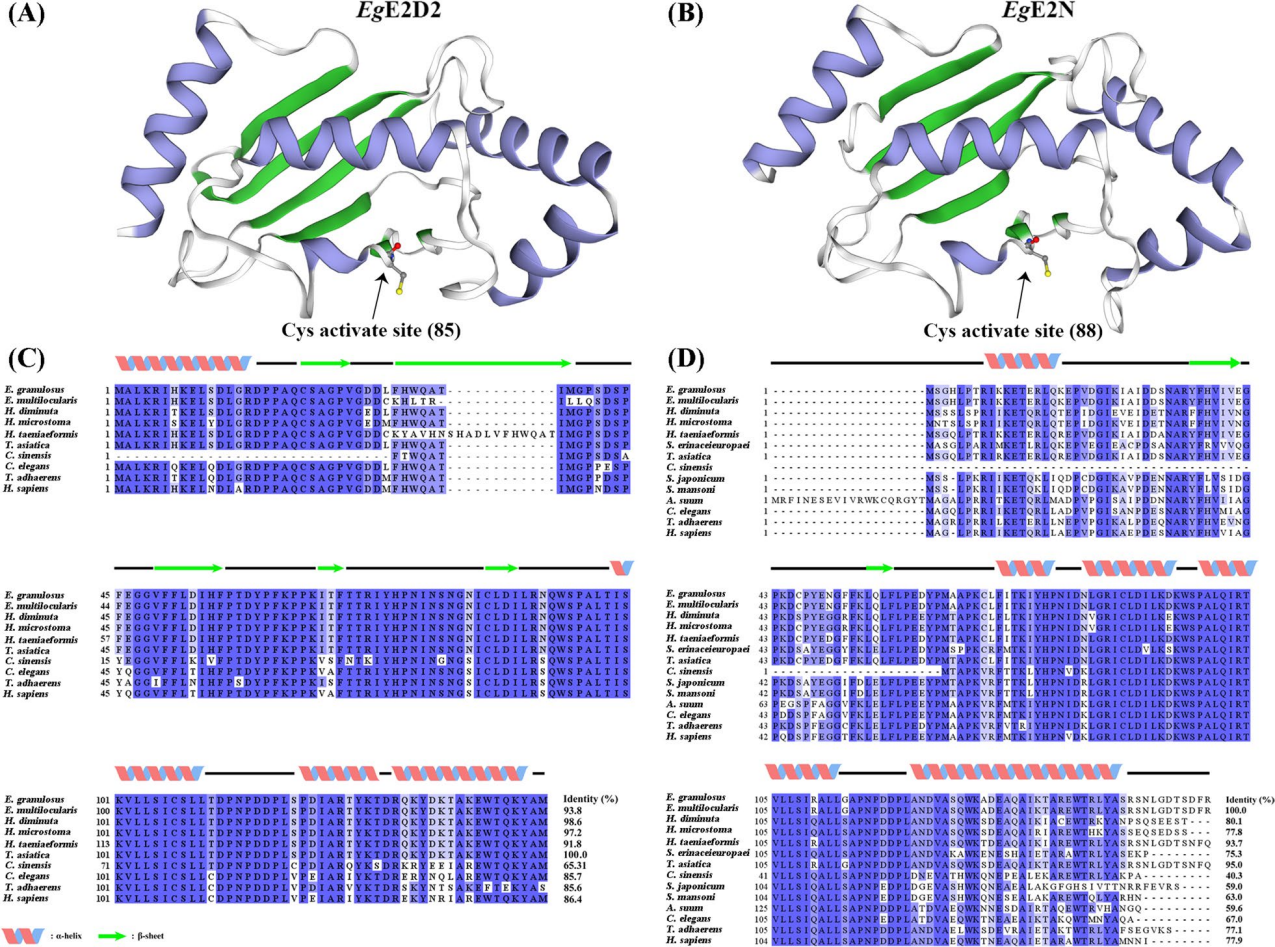
The three-dimensional (3D) structure of *Eg*E2D2 **A** and *Eg*E2N **B** and multiple sequences alignment of *Eg*E2D2 **C** and *Eg*E2N **D**. *E. granulosus* (NCBI: QEO33306.1); *E. multilocularis* (UniProt: A0A087VYI0); *H. diminuta* (UniProt: A0A0R3SUP0); *H. microstoma* (NCBI: CDS29458.1); *H. taeniaeformis* (NCBI: VDM16792.1); *T. asiatica* (NCBI: VDK24545.1); *C. sinensis* (NCBI: GAA57084.1); *C. elegans* (UniProt: P35129); *T. adhaerens* (UniProt: B3S643); *H. sapiens* (UniProt: P62837). (B) *E. granulosus* (NCBI: XP_024351120.1); *E. multilocularis* (NCBI: CDS35641.1); *H. diminuta* (NCBI: VUZ44905.1); *H. microstoma* (NCBI: CDS26822.1); *H. taeniaeformis* (NCBI: VDM34754.1); *S. erinaceieuropaei* (NCBI: VZI31528.1); *T. asiatica* (NCBI: VDK31863.1); *C. sinensis* (UniProt: G7YFT3); *S. japonicum* (UniProt: A0A4Z2DGS1); *S. mansoni* (UniProt: Q2MK73); *A. suum* (UniProt: F1KVF0); *C. elegans* (UniProt: Q95XX0); *T. adhaerens* (NCBI: XP_002111946.1); *H. sapiens* (UniProt: P61088). Identical amino acid residues are shaded blue; the darker the color, the more conservative the sequence

The basic physical and chemical properties of *Eg*E2D2 (kDa = 16) and *Eg*E2N (kDa = 18) are presented in Table 2. The prediction of secondary structure and tertiary structure (Fig. 1A, B) revealed that they were similar in spatial structure, containing four α-helices and four β-folds, forming the UBC domain of E2. Both *Eg*E2D2 and *Eg*E2N belong to class I, indicating that their genetic biological function mainly depends on the UBC domain.

**Table 2:** Basic physicochemical properties of *Eg*E2D2 and *Eg*E2N

	ORF (bp)	Amino acid (aa)	Molecular weight (kDa)	Isoelectric point (pI)	Signal peptide/transmembrane domain	Subcellular location	Active site (Cys)	Classification
*Eg*E2D2	444	147	16	6.39	–/–	N	85	I
*Eg*E2N	486	161	18	6.83	–/–	C, N	88	I

*C* cytoplasm, *N* nucleus

Sequence alignment (Fig. 1C, D) showed that *Eg*E2D2 shared 100.0% identity with *Taenia asiatica* (*T. asiatica*) E2D2, and has 98.6%, 97.2%, 93.8%, and 91.8% similarity with *Hymenolepis diminuta* (*H. diminuta*) E2D2, *Hymenolepis microstoma* (*H. microstoma*) E2D2, *Echinococcus multilocularis* (*E. multilocularis*) E2D2, and *Hydatigera taeniaeformis* (*H. taeniaeformis*) E2D2. *Eg*E2N shared 100.0% identity with E2N of *E. multilocularis*, by contrast, its homology compared with *T. asiatica* and *H. taeniaeformis* is 95.0% and 93.7%, respectively.

The phylogenetic analysis (Additional file 3: Fig. S3) showed that *Eg*E2D2 and *Eg*E2N are grouped together with *E. multilocularis*, *T. asiatica*, *H. microstoma*, and *H. taeniaeformis*, indicating their close evolutionary relationship. By contrast, *Eg*E2D2 and *Eg*E2N showed a distinct separation from trematodes and nematodes, and are more distantly related to mammals.

### Expression, purification, and Western blotting of r*Eg*E2D2 and r*Eg*E2N

**Fig. 2:**
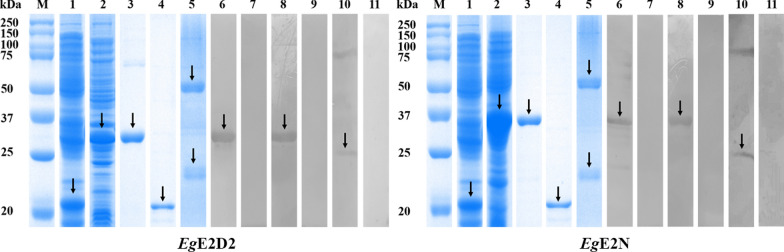
Expression, purification, and Western blotting of r*Eg*E2D2 and r*Eg*E2N. M: Protein marker; (1) Protein of pET32a (+); (2) r*Eg*E2D2/r*Eg*E2N crude protein; (3) Purified r*Eg*E2D2 and r*Eg*E2N; (4) Purified pET32a (+) protein; (5) Purified r*Eg*E2D2-IgG and r*Eg*E2N-IgG; (6) Purified recombinant protein probed with rat polyclonal antibody; (7) Purified recombinant protein probed with pre-immunized rat serum; (8) Purified recombinant protein probed with serum from sheep naturally infected with *E. granulosus*; (9) Purified recombinant protein probed with non-infected sheep serum; (10) Total protein of PSCs probed with rat polyclonal antibody; (11) Total protein of PSCs probed with pre-immunized rat serum

As expected, the molecular weights of r*Eg*E2D2 and r*Eg*E2N were about 34 kDa and 36 kDa, respectively (Fig. 2: Lanes 1, 2, 3, and 4). It should be noted that the pET-32a(+) tag proteins weigh about 18 kDa. Both proteins were expressed in a soluble protein. The purified antiserum IgG exhibited a heavy chain of 50 kDa and a light chain of 23 kDa (Fig. 2: Lane 5). Western blotting showed that both r*Eg*E2D2 and r*Eg*E2N could be specifically recognized by antiserum and sheep positive serum (Fig. 2: Lanes 6 and 8), indicating their strong immunoreactivity. In addition, anti-r*Eg*E2D2 IgG and anti-r*Eg*E2N IgG successfully recognized the natural protein in PSCs (Fig. 2: Lane 10). No bands appeared when the recombinant proteins were incubated with rat serum IgG, sheep negative serum, and PSCs crude protein incubated with rat serum IgG (Fig. 2: Lanes 7, 9, and 11).

### Immunolocalization and secretory analysis of EgE2D2 and EgE2N

**Fig. 3:**
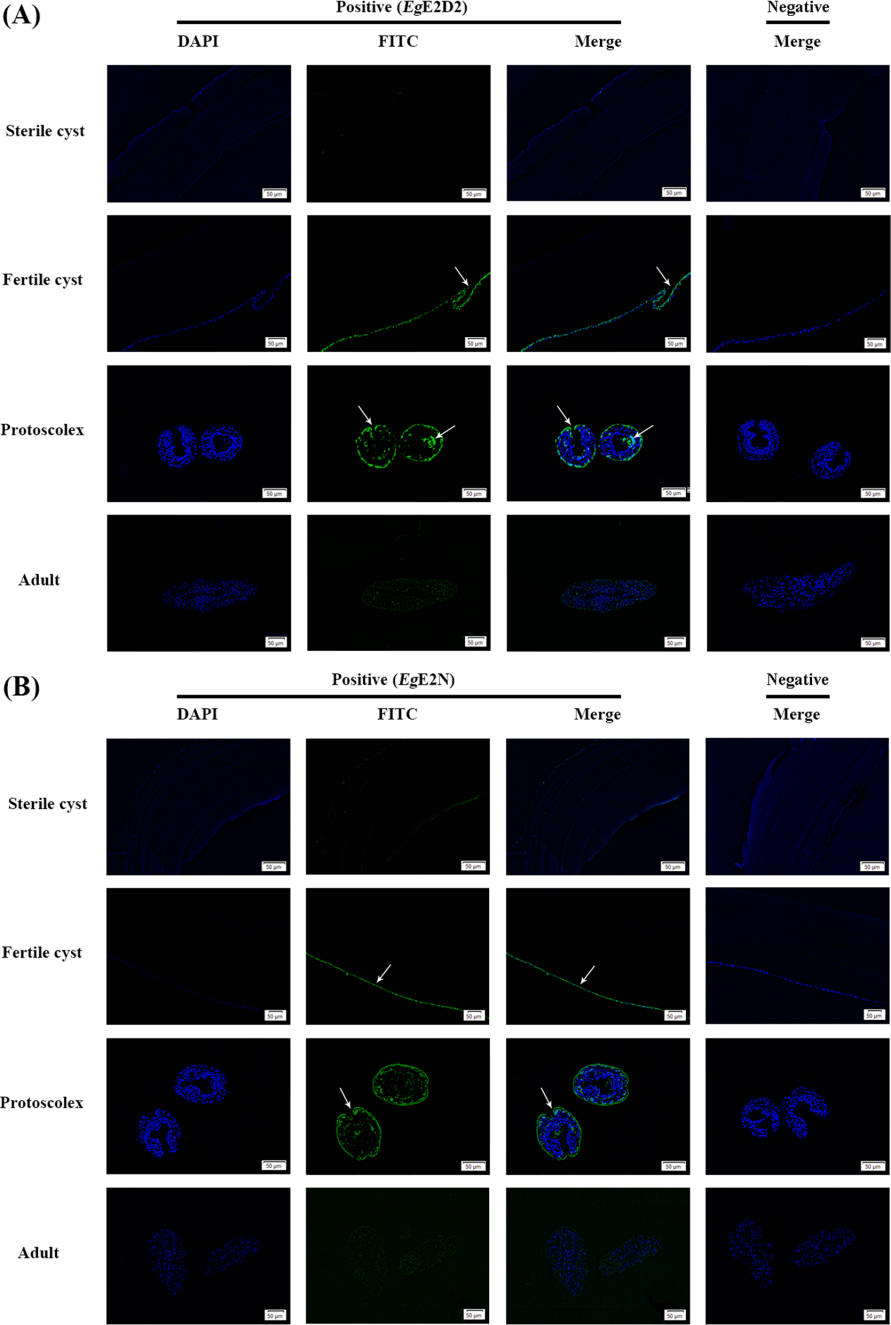
Immunolocalization of *Eg*E2D2 **A** and *Eg*E2N **B** (×200)

*Eg*E2D2 and *Eg*E2N exhibited a wide distribution in the germinal layer of the fertile cysts, mainly in the epidermis and hook in PSCs, while limited green fluorescence was observed in the sterile cysts. Notably, there were distinct differences in the distribution patterns of *Eg*E2D2 and *Eg*E2N in 25-day strobilated worms compared with PSCs. In 25-day strobilated worms, the fluorescence intensity of both *Eg*E2D2 and *Eg*E2N was weak and scattered (Fig. 3A, B).

**Fig. 4:**
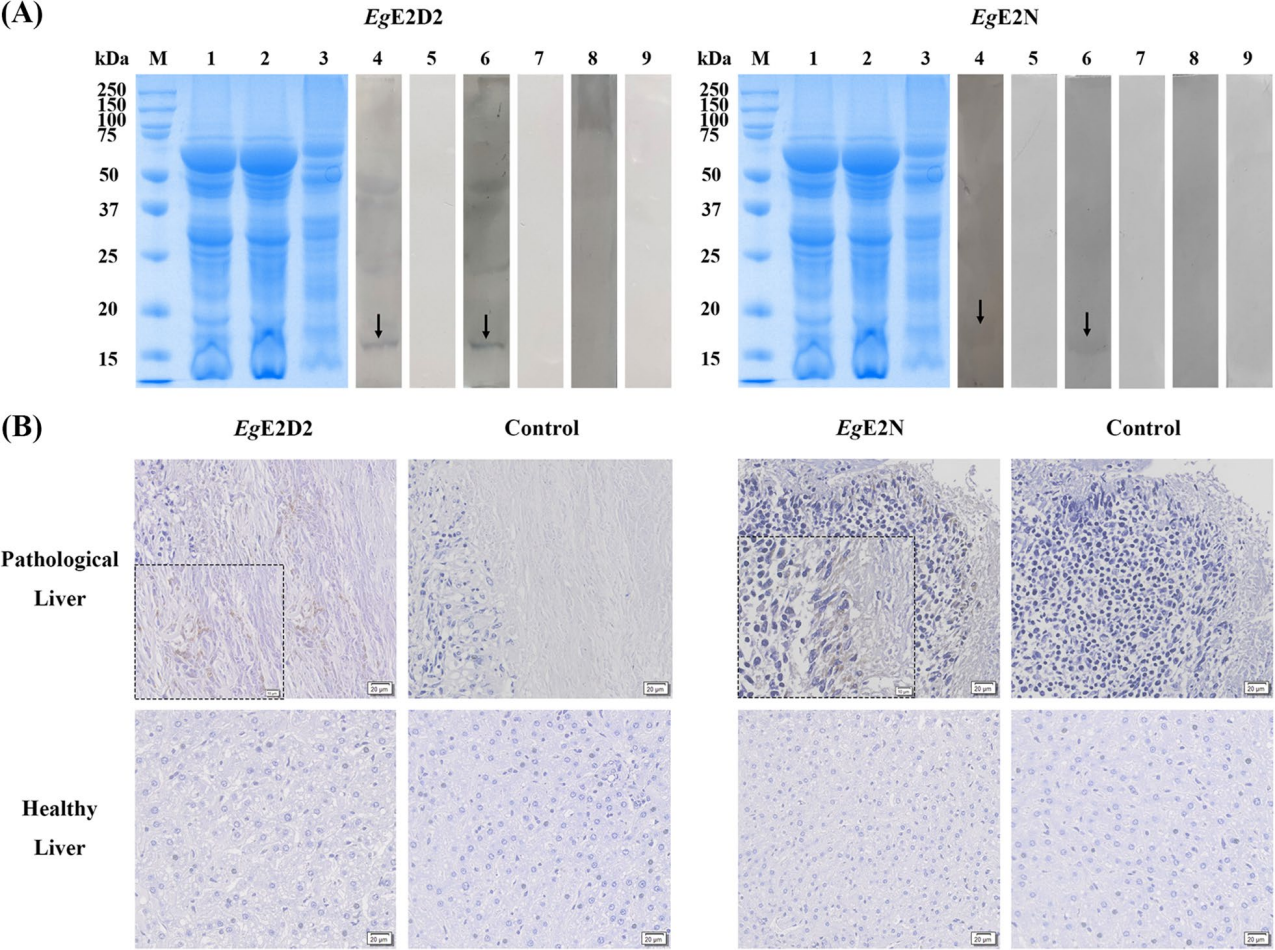
Analysis of secretory characteristics of *Eg*E2D2 and *Eg*E2N. **A** Western blotting analysis. M: Protein marker; (1) Fresh HF; (2) HF without *Eg*sEV; (3) *Eg*sEV; (4) Fresh HF probed with rat polyclonal antibody; (5) Fresh HF probed with pre-immunized rat serum; (6) HF without *Eg*sEV probed with rat polyclonal antibody; (7) HF without *Eg*sEV probed with pre-immunized rat serum; (8) *Eg*sEV probed with rat polyclonal antibody; (9) *Eg*sEV probed with pre-immunized rat serum. **B** Immunohistochemistry was used to analyze whether *Eg*E2D2 and *Eg*E2N were secreted into sheep liver (×200/×400)

Considering that *Eg*E2D2 and *Eg*E2N lack signal peptides for the classical secretory pathway, we further investigated whether they are vesicular structure-mediated secretion [26]. Transmission electron microscopy and nanoparticle tracking analysis showed that extracellular vesicles originating from HF exhibited a typical cup-shaped structure with sizes ranging from 74 nm to 184 nm (Additional file 2: Figure. S2), consistent with the characteristics of sEV. Western blotting demonstrated distinct bands in both fresh HF and HF without *Eg*sEV, while no band was observed in *Eg*sEV (Fig. 4A: Lines 4–9). Immunolocalization of *Eg*E2D2 and *Eg*E2N in the liver tissue of CE-infected sheep showed a strong brown signal at the junction of liver tissue and fibrous layer. By contrast, no positive signal was detected in the healthy liver tissue or negative control. These results indicated that *Eg*E2D2 and *Eg*E2N could be secreted into the liver tissue through a non-classical secretory pathway, possibly excluding small extracellular vesicles (Fig. 4B).

### r*Eg*E2D2 and r*Eg*E2N have ubiquitin binding activity

**Fig. 5:**
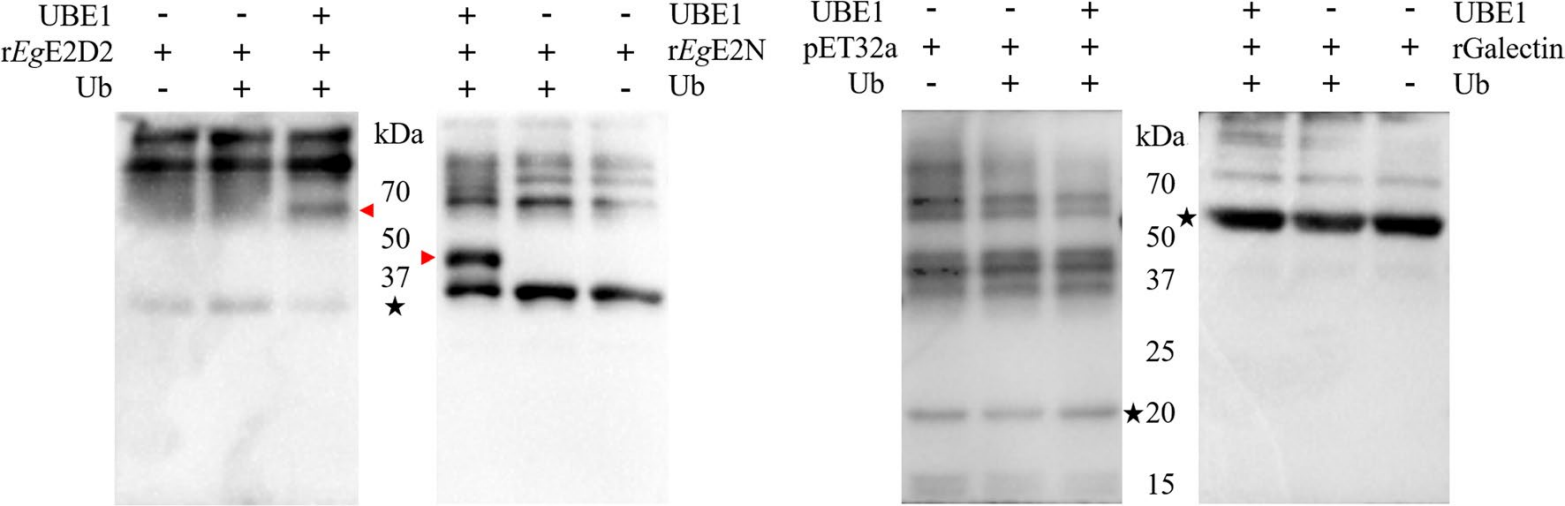
Detection of r*Eg*E2D2 and r*Eg*E2N enzyme activity. The red triangles indicate that the bands are mono-ubiquitinated or poly-ubiquitinated E2s. The asterisks indicate that the bands are unreacted recombinant proteins, and the other bands are impurity proteins

The molecular sizes of r*Eg*E2D2, r*Eg*E2N, pET32a, and Galectin were 34 kDa, 36 kDa, 18 kDa, and 52 kDa, respectively. The results (Fig. 5) showed that the polymer bands formed by the combination of r*Eg*E2D2 or r*Eg*E2N with multiple Ub were detected in the reaction solution with the coexistence of UBE1, *Eg*E2, and Ub. pET32a and rGalectin did not form such polymer bands, indicating that r*Eg*E2D2 and r*Eg*E2N have Ub conjugating activity.

### Toxicity analysis of recombinant proteins to LX-2 cells

**Fig. 6:**
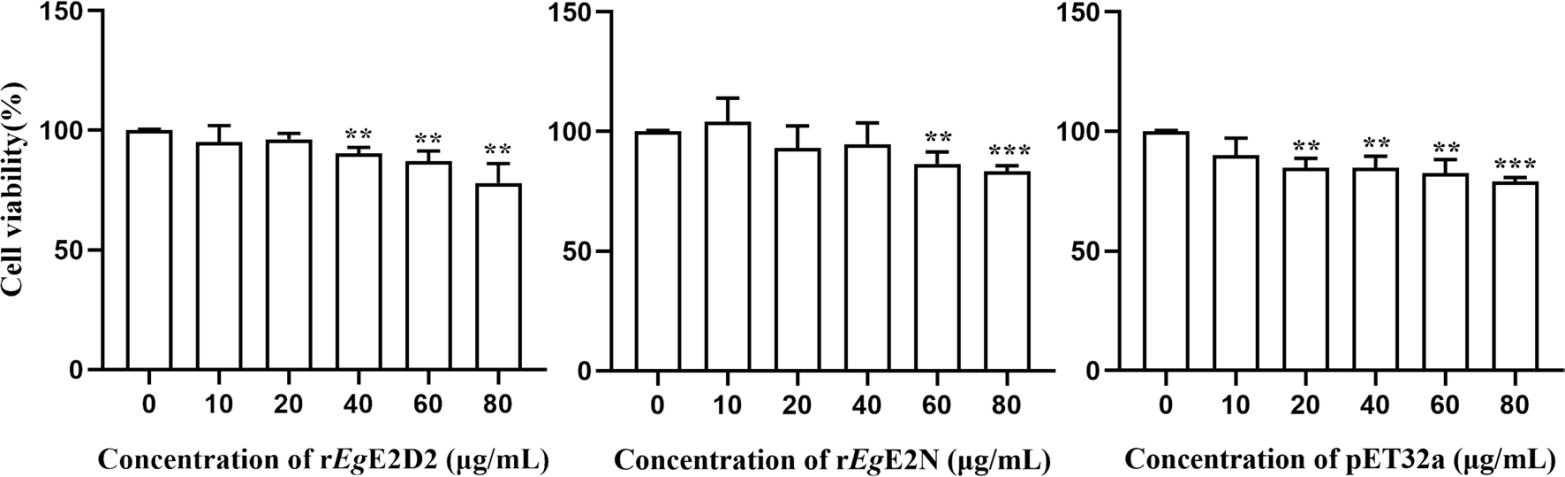
Cell viability after treatment with r*Eg*E2D2, r*Eg*E2N, and pET32a. ‘*’ is the expression level of treatment group compared with control group

After removing endotoxin from the recombinant proteins, the concentration of endotoxin was determined to be less than 0.01 EU/mL. To ensure subtoxic doses of the recombinant proteins, LX-2 cells were incubated with protein concentrations ranging from 0 μg/mL to 80 μg/mL for 24 h, and cytotoxic effects were evaluated by CCK-8 assay. As shown in Fig. 6, tolerated concentrations of cells were r*Eg*E2D2 (10, 20 μg/mL) and r*Eg*E2N (10, 20, 40 μg/mL), respectively. Moreover, higher concentrations of these proteins, as well as pET32a at 20 μg/mL, will also significantly inhibit cell growth.

### r*Eg*E2D2 and r*Eg*E2N promote the expression of fibrogenic genes in TGFβ1-induced LX-2

**Fig. 7:**
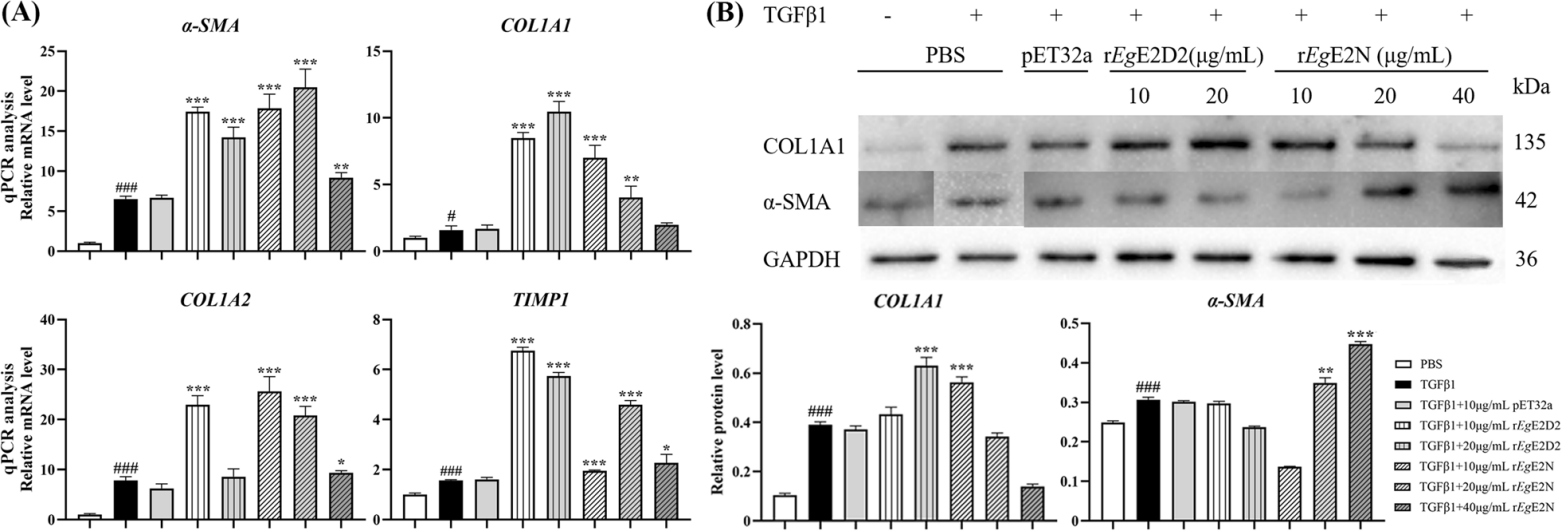
**A** qRT-PCR was used to detect the expression of pro-fibrogenic genes in LX-2 cells. ‘#’ is the expression level of the TGFβ1 group compared with the PBS group. **B** Western blotting was used to detect the expression of pro-fibrogenic genes in LX-2 cells

qRT-PCR results showed that TGFβ1 significantly increased the expression of *α-SMA*, *COL1A1*, *COL1A2*, and *TIMP1* in LX-2 cells compared with the PBS group (Fig. 7A). Compared with the TGFβ1 treatment group, the addition of 10 μg/mL r*Eg*E2D2, 10 μg/mL r*Eg*E2N, and 20 μg/mL r*Eg*E2N resulted in a significant increase in the expression of four fibrogenic genes. Additionally, the presence of 20 μg/mL r*Eg*E2D2 could significantly upregulate the expression of three genes except for *COL1A2*.

Western blotting showed that with the addition of TGFβ1, the protein expression of *COL1A1* was significantly increased in the presence of 20 μg/mL r*Eg*E2D2 or 10 μg/mL r*Eg*E2N, whereas the expression of *α-SMA* was only increased in the r*Eg*E2N groups at the concentrations of 20 μg/mL or 40 μg/mL (Fig. 7B). There was no significant difference in protein expression between the pET32a treated group and TGFβ1 group, indicating that specific concentrations of r*Eg*E2D2 and r*Eg*E2N could promote the protein expression of fibrosis-related genes in TGFβ1-induced LX-2 cells.

### r*Eg*E2D2 and r*Eg*E2N promote the proliferation and migration of TGFβ1-induced LX-2

**Fig. 8:**
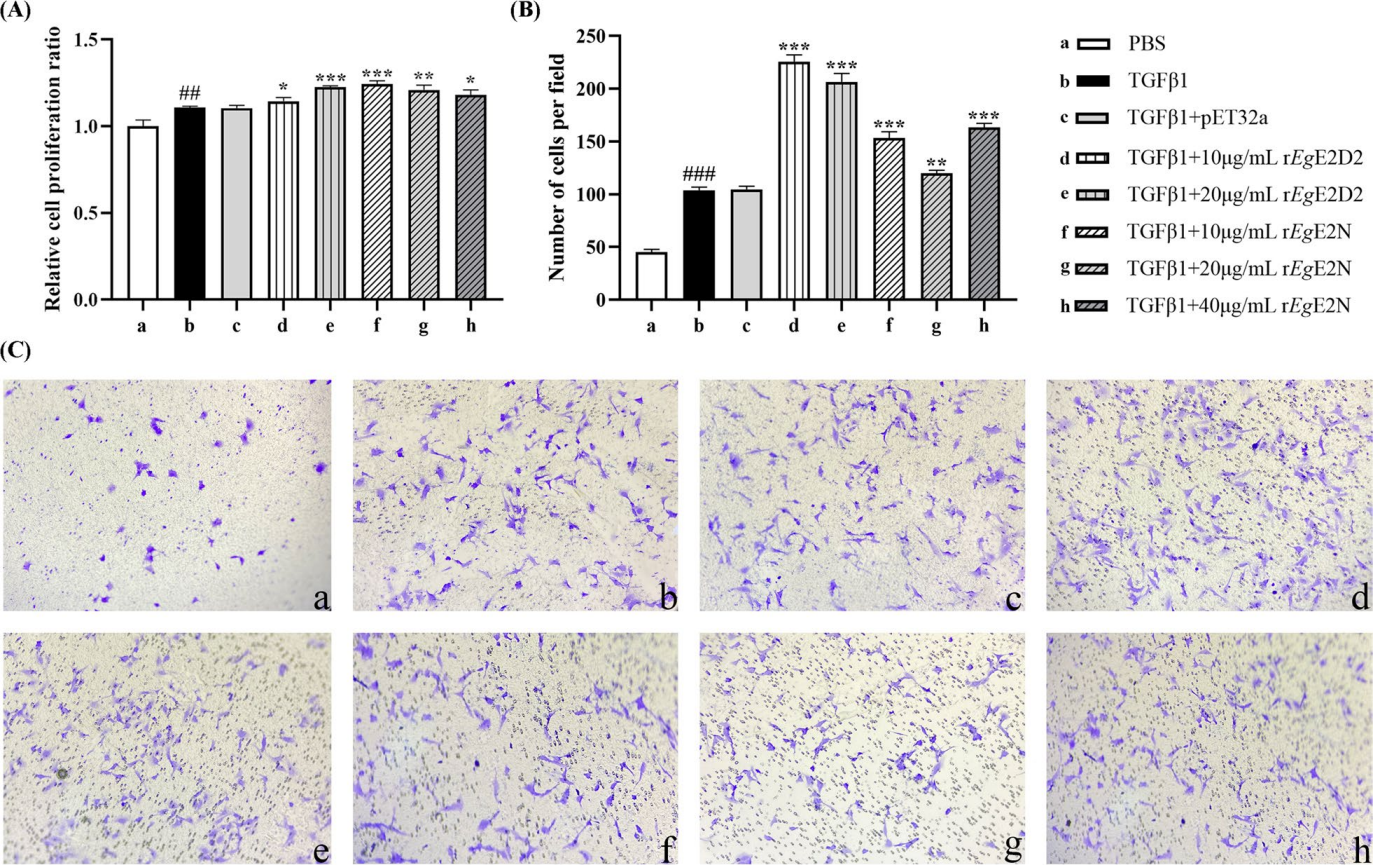
Effects of r*Eg*E2D2 and r*Eg*E2N on proliferation **A** and migration **B**&**C** of TGFβ1-induced LX-2 cells

The proliferation and migration of activated hepatic stellate cells (HSCs) play an important role in the development of hepatic fibrosis [27]. Therefore, we used the CCK-8 assay to investigate the effects of r*Eg*E2D2 and r*Eg*E2N on the proliferation and migration of TGFβ1-induced LX-2 cells. Compared with the control group (Fig. 8A), r*Eg*E2D2 and r*Eg*E2N significantly promoted the proliferation of LX-2 cells induced by TGFβ1, whereas pET32a protein did not exhibit such effects. The chemotactic potential of r*Eg*E2D2 and r*Eg*E2N on TGFβ1-induced LX-2 cells was further investigated by using 24-well transwell plates. Compared with the PBS group, TGFβ1 treatment significantly increased the number of LX-2 cells migrating to the lower chamber (Fig. 8B, C). Importantly, compared with the TGFβ1 treatment group, both r*Eg*E2D2 and r*Eg*E2N significantly promote the migration of TGFβ1-induced LX-2 cells.

## Discussion

Ubiquitin-conjugating enzyme plays a central role in the ubiquitin–proteasome pathway: it receives the activated Ub at its front end and subsequently transfers the Ub to the target protein through ubiquitin ligase, thereby modulating the stability and activity of the target protein [28]. Most E2 enzymes possess a UBC domain that consists of approximately 150–200 amino acids, encompassing four α-helices and four β-folds [9]. The enzyme activity of E2 is dependent on the active site cysteine residue, which facilitates the binding and transfer of Ub [29]. Our findings demonstrate that *Eg*E2D2 and *Eg*E2N exhibit conserved amino acid sequences and tertiary structures, including intact UBC domains and active sites. These observations suggest that their biological functions may be similar to those found in mammals [30]. Furthermore, the ubiquitin-binding activity of r*Eg*E2D2 and r*Eg*E2N was confirmed by the ubiquitination experiment, indicating the recombinant molecules exhibit certain biological functions in vitro. Additionally, our study revealed the specific recognition of both r*Eg*E2D2 and r*Eg*E2N by CE-infected sheep serum, highlighting their strong reactivity and potential as diagnostic antigens.

Our results of immunofluorescence localization showed that *Eg*E2D2 and *Eg*E2N were widely distributed in the germinal layer of fertile cyst, epidermis, and hook of PSCs. Notably, the epidermis of tapeworm serves as a tissue for absorption of host nutrient, waste excretion, and signal transduction, which indicates that *Eg*E2D2 and *Eg*E2N may participate in these processes, and thereby contribute to the growth and development of PSCs [31]. Proteomic data have shown that both EgE2D2 and EgE2N are present in sheep-derived HF EV [16, 17], and we further confirmed that *Eg*E2D2 and *Eg*E2N were detected in sheep-derived HF while not in the sEV. This phenomenon may indicate that EgE2D2 and EgE2N were secreted through other EV subtypes such as large EV or exosomes. Immunofluorescence localization showed that *Eg*E2D2 and *Eg*E2N were mostly distributed in germinal layer and PSCs epidermis, providing favorable conditions for their secretion into HF. Furthermore, our immunohistochemistry results showed that *Eg*E2D2 and *Eg*E2N could enter the junction of liver tissue and fibrous layer after secretion, and their specific function and secretion pathway need to be explored in the future.

α-SMA is considered a marker of myofibroblasts, which exhibit wide distribution in muscle cells, and its expression regulates muscle contractile function. COL1A1 and COL3A1 are crucial components of ECM involved in cell migration, differentiation, and reflecting the process of fibrosis [32]. TIMP1, which is secreted by HSCs, could inhibit the activity of matrix metalloproteinases, thereby blocking the degradation of collagen and promoting fibrosis formation [33]. These fibrogenic genes play an essential role in reflecting the fibrosis progression, as evidenced by the overall increasing trend in their expression levels in TGFβ1-induced LX-2 cells on the basis of qRT-PCR experiment. Furthermore, higher expression levels of fibrogenic genes were observed in TGFβ1-induced LX-2 cells with the addition of rEgE2D2 or rEgE2N. However, when considering the results of Western blotting experiments, the differential expression of α-SMA and COL1A1 proteins may be attributed to rEgE2D2 or rEgE2N degrading excessive amounts of proteins necessary for normal cellular function. As for cellular function, both r*Eg*E2D2 and r*Eg*E2N could promote the proliferation and migration of TGFβ1-induced LX-2 cells, indicating that the entry of *Eg*E2D2 and *Eg*E2N into the liver tissue facilitated hepatic fibrosis development. Extensive adventitial layer fibrosis provides mechanical support for fluid-filled cysts, which is usually considered as a mechanism by which the host restricts the growth and infection of metacestode. However, recent studies conducted in experimental mice have shown that IL-17A could reduce fibrosis, resulting in a reduction in parasite numbers [34, 35]. This evidence suggests that fibrotic response is related to the survival of parasites, and the regulation of fibrosis formation by specific proteins such as *Eg*E2D2 and *Eg*E2N is necessary for the development of hydatid cysts.

It was reported that HF could significantly promote the expression of α-SMA, COL1A1, and the proliferation of LX-2 cells, indicating that the parasite components in HF participated in the development of liver fibrosis by activating HSCs [36]. Our study further demonstrated that *Eg*E2D2 and *Eg*E2N are secreted into the HF and participate in the process of liver fibrosis by enhancing the expression of fibrosis-related genes and promoting the growth as well as migration of LX-2 cells. Ubiquitination is considered to be an important regulator of TGFβ signaling pathway by regulating Smads, and the specific mechanism of TGFβ1 activation in LX-2 cells involves the function of E2s: the activated TGFβ1 signal is transferred to Smads protein through homologous receptors, thus enhancing the transcription of fiber-forming genes [37, 38]. On the basis of this information, we speculate that *Eg*E2D2 and *Eg*E2N may promote fibrosis by promoting the signal transduction of TGFβ1. It is important to note that the interaction and mutual regulation among the ECM, cells, and cytokines in liver fibrosis formation are highly complex. Further studies are required to explore the specific mechanisms through which EgE2D2 and EgE2N promote liver fibrosis in TGFβ1-induced LX-2 cells.

### Supplementary Information

**Additional file 1: Figure S1.** The HE staining of samples. (A): Sterile cyst, fertile cyst, PSC, and 25-day strobilated worm (HE × 200); (B): Fertile cyst and surrounding liver tissue (HE × 400); (C): Healthy liver tissue (HE × 200). Abbreviations: G, germinal layer; H, hooks; S, suckers; Sc, scolex.

**Additional file 2: Figure S2. **Transmission electron microscopy (A) and nanoparticle tracking analysis (B) of extracellular vesicles originating from HF.

**Additional file 3: Figure S3. **The phylogenetic tree (ML) of *Eg*E2D2 and *Eg*E2N.

## Data Availability

The data supporting the findings of the study must be available within the article and/or its supplementary materials, or deposited in a publicly available database.

## Abbreviations

CE: Cystic echinococcosis
E2s: Ubiquitin-conjugating enzymes
*E*. *granulosus*: *Echinococcus* *granμlosus*
PSCs: Protoscoleces
Ub/Ubl: Ubiquitin/ubiquitin-like
HF: Hydatid fluid
EV: Extracellular vesicles
CCK-8: Cell counting Kit-8
HE staining: Hematoxylin–eosin staining
ML: Maximum likelihood
UBC domain: Ubiquitin-conjugating domain
ECM: Extracellular matrix
HSC: Hepatic stellate cells

## References

[1] Deplazes P, Rinaldi L, Alvarez Rojas CA, Torgerson PR, Harandi MF, Romig T (2017). Global distribution of alveolar and cystic echinococcosis. Adv Parasitol.

[2] Wen H, Vuitton L, Tuxun T, Li J, Vuitton DA, Zhang W (2019). Echinococcosis: advances in the 21st century. Clin Microbiol Rev.

[3] World Health Organization & Food and Agriculture Organization of the United Nations (2014). Multicriteria-based ranking for risk management of food-borne parasites: report of a Joint FAO/WHO expert meeting, 3–7 September 2012.

[4] World Health Organization (2020). Ending the neglect to attain the sustainable development goals: a road map for neglected tropical diseases 2021–2030.

[5] Thompson RCA (2017). Biology and systematics of *Echinococcus*. Adv Parasitol.

[6] Hidalgo C, Stoore C, Strull K, Franco C, Corrêa F, Jiménez M (2019). New insights of the local immune response against both fertile and infertile hydatid cysts. PLoS ONE.

[7] Vuitton DA (2003). The ambiguous role of immunity in echinococcosis: protection of the host or of the parasite?. Acta Trop.

[8] van Wijk SJ, Timmers HT (2010). The family of ubiquitin-conjugating enzymes (E2s): deciding between life and death of proteins. FASEB J.

[9] Stewart MD, Ritterhoff T, Klevit RE, Brzovic PS (2016). E2 enzymes: more than just middle men. Cell Res.

[10] Liu W, Tang X, Qi X, Fu X, Ghimire S, Ma R (2020). The ubiquitin conjugating enzyme: an important ubiquitin transfer platform in ubiquitin-proteasome system. Int J Mol Sci.

[11] Hosseini SM, Okoye I, Chaleshtari MG, Hazhirkarzar B, Mohamadnejad J, Azizi G (2019). E2 ubiquitin-conjugating enzymes in cancer: implications for immunotherapeutic interventions. Clin Chim Acta.

[12] Meyer HJ, Rape M (2014). Enhanced protein degradation by branched ubiquitin chains. Cell.

[13] Kumari R, Gupta P, Tiwari S (2018). Ubc7/Ube2g2 ortholog in *Entamoeba **histolytica*: connection with the plasma membrane and phagocytosis. Parasitol Res.

[14] Rojas F, Koszela J, Búa J, Llorente B, Burchmore R, Auer M (2017). The ubiquitin-conjugating enzyme CDC34 is essential for cytokinesis in contrast to putative subunits of a SCF complex in
*Trypanosoma
brucei*. PLoS Negl Trop Dis.

[15] White RR, Ponsford AH, Weekes MP, Rodrigues RB, Ascher DB, Mol M (2016). Ubiquitin-dependent modification of skeletal muscle by the parasitic nematode,
*Trichinella
spiralis*. PLoS Pathog.

[16] Zhou X, Wang W, Cui F, Shi C, Ma Y, Yu Y (2019). Extracellular vesicles derived from
*Echinococcus
granulosus* hydatid cyst fluid from patients: isolation, characterization and evaluation of immunomodulatory functions on T cells. Int J Parasitol.

[17] Siles-Lucas M, Sánchez-Ovejero C, González-Sánchez M, González E, Falcón-Pérez JM, Boufana B (2017). Isolation and characterization of exosomes derived from fertile sheep hydatid cysts. Vet Parasitol.

[18] Cizmar P, Yuana Y (2017). Detection and characterization of extracellular vesicles by transmission and cryo-transmission electron microscopy. Methods Mol Biol.

[19] Bachurski D, Schuldner M, Nguyen PH, Malz A, Reiners KS, Grenzi PC (2019). Extracellular vesicle measurements with nanoparticle tracking analysis—An accuracy and repeatability comparison between NanoSight NS300 and ZetaView. J Extracell Vesicles.

[20] Zhan J, Song H, Wang N, Guo C, Shen N, Hua R (2020). Molecular and functional characterization of inhibitor of apoptosis proteins (IAP, BIRP) in
*Echinococcus granulosus*. Front Microbiol.

[21] Zhao Q, Tian M, Li Q, Cui F, Liu L, Yin B (2013). A plant-specific in vitro ubiquitination analysis system. Plant J.

[22] Nguyen PT, Kanno K, Pham QT, Kikuchi Y, Kakimoto M, Kobayashi T (2020). Senescent hepatic stellate cells caused by deoxycholic acid modulates malignant behavior of hepatocellular carcinoma. J Cancer Res Clin Oncol.

[23] Cheng Q, Li C, Yang C, Zhong Y, Wu D, Shi L (2019). Methyl ferulic acid attenuates liver fibrosis and hepatic stellate cell activation through the TGF-β1/Smad and NOX4/ROS pathways. Chem Biol Interact.

[24] He D, Zhang X, Liu K, Pang R, Zhao J, Zhou B (2014). In vitro inhibition of the replication of classical swine fever virus by porcine Mx1 protein. Antiviral Res.

[25] Zhou L, Shang M, Shi M, Zhao L, Lin Z, Chen T (2016). *Clonorchis
sinensis* lysophospholipase inhibits TGF-β1-induced expression of pro-fibrogenic genes through attenuating the activations of Smad3, JNK2, and ERK1/2 in hepatic stellate cell line LX-2. Parasitol Res.

[26] Zhang M, Liu L, Lin X, Wang Y, Li Y, Guo Q (2020). A translocation pathway for vesicle-mediated unconventional protein secretion. Cell.

[27] Chiu Y, Wei C, Lin Y, Hsu Y, Chang M (2014). IL-20 and IL-20R1 antibodies protect against liver fibrosis. Hepatology.

[28] Du X, Song H, Shen N, Hua R, Yang G (2021). The molecular basis of ubiquitin-conjugating enzymes (E2s) as a potential target for cancer therapy. Int J Mol Sci.

[29] Zhang W, Zhuang Y, Zhang Y, Yang X, Zhang H, Wang G (2017). Uev1A facilitates osteosarcoma differentiation by promoting Smurf1-mediated Smad1 ubiquitination and degradation. Cell Death Dis.

[30] Costa MP, Oliveira VF, Pereira RV, de Abreu FC, Jannotti-Passos LK, Borges WC (2015). In silico analysis and developmental expression of ubiquitin-conjugating enzymes in
*Schistosoma mansoni*. Parasitol Res.

[31] Jones MK, Gobert GN, Zhang L, Sunderland P, McManus DP (2004). The cytoskeleton and motor proteins of human schistosomes and their roles in surface maintenance and host-parasite interactions. BioEssays.

[32] Kim KK, Sheppard D, Chapman HA (2018). TGF-β1 signaling and tissue fibrosis. Cold Spring Harb Perspect Biol.

[33] Bonnans C, Chou J, Werb Z (2014). Remodelling the extracellular matrix in development and disease. Nat Rev Mol Cell Biol.

[34] Labsi M, Soufli I, Khelifi L, Amir ZC, Touil-Boukoffa C (2018). In vivo treatment with IL-17A attenuates hydatid cyst growth and liver fibrogenesis in an experimental model of echinococcosis. Acta Trop.

[35] Tamarozzi F, Mariconti M, Neumayr A, Brunetti E (2016). The intermediate host immune response in cystic echinococcosis. Parasite Immunol.

[36] Zhang C, Wang L, Ali T, Li L, Bi X, Wang J (2016). Hydatid cyst fluid promotes peri-cystic fibrosis in cystic echinococcosis by suppressing miR-19 expression. Parasit Vectors.

[37] Yoshida K, Matsuzaki K (2012). Differential regulation of TGF-β/Smad signaling in hepatic stellate cells between acute and chronic liver injuries. Front Physiol.

[38] Soond SM, Chantry A (2011). How ubiquitination regulates the TGF-β signalling pathway: new insights and new players: new isoforms of ubiquitin-activating enzymes in the E1–E3 families join the game. BioEssays.

