# Supplementary figures and images for: Echinococcus granulosus ubiquitin-conjugating enzymes (E2D2 and E2N) promote the formation of liver fibrosis in TGFβ1-induced LX-2 cells
Source: Parasit Vectors. 2024 Apr 20;17:190. doi: 10.1186/s13071-024-06222-8 (PMC11031992; doi:10.1186/s13071-024-06222-8)

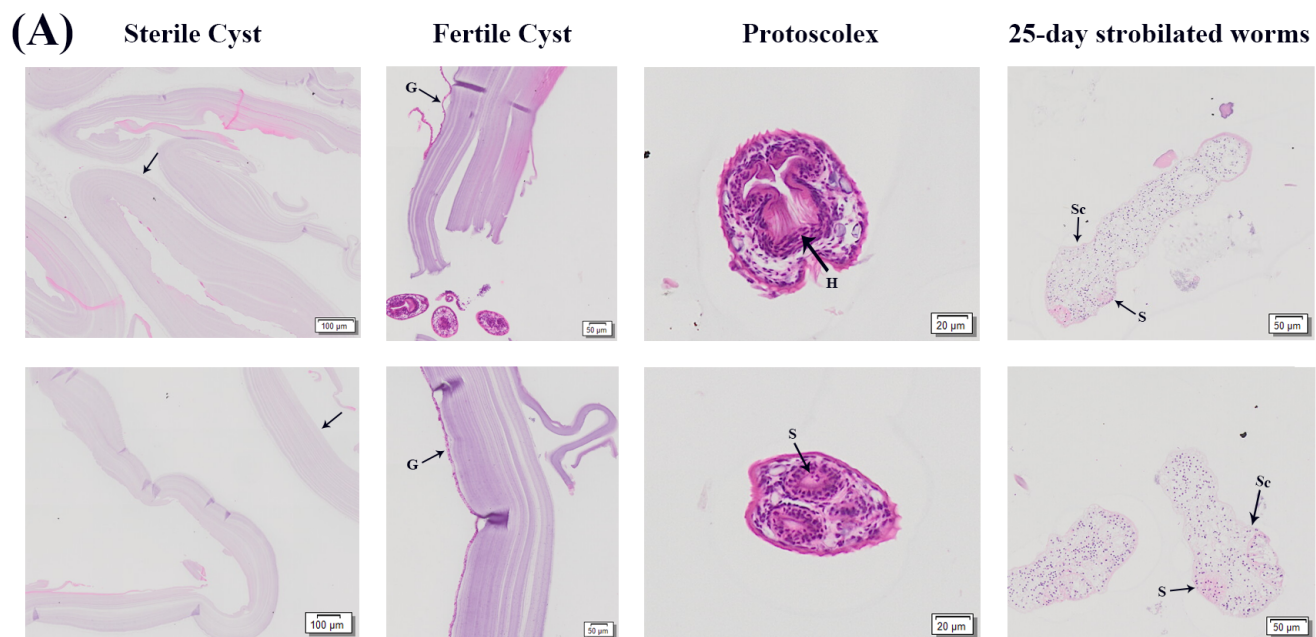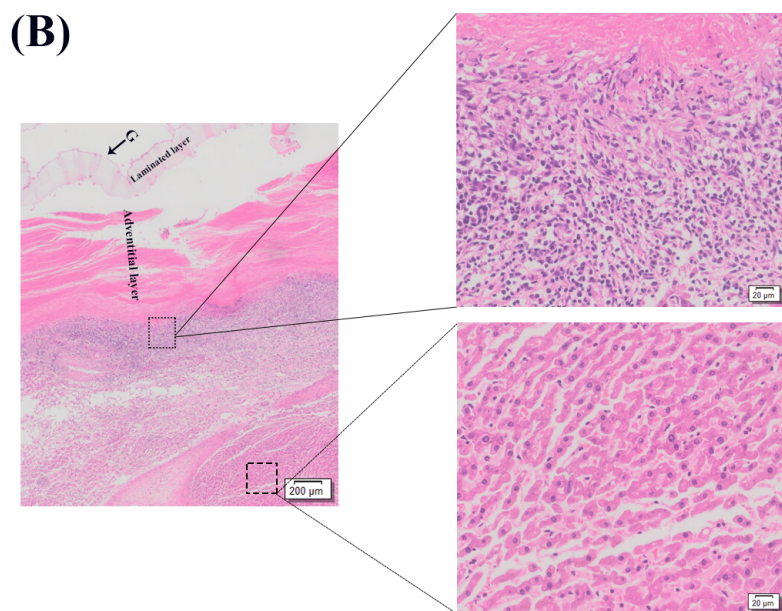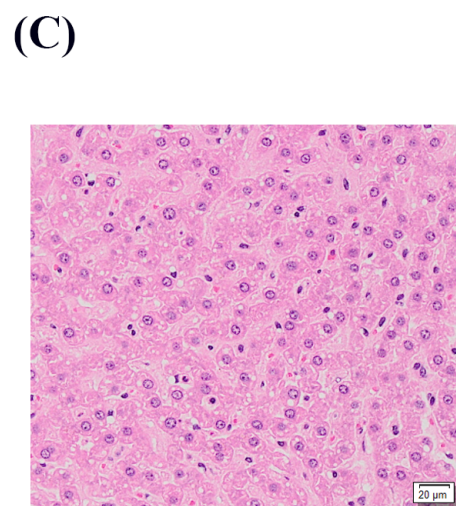

Supplement: Supplementary file 1 — Additional file 1: Figure S1. The HE staining of samples. (A): Sterile cyst, fertile cyst, PSC, and 25-day strobilated worm (HE × 200); (B): Fertile cyst and surrounding liver tissue (HE × 400); (C): Healthy liver tissue (HE × 200). Abbreviations: G, germinal layer; H, hooks; S, suckers; Sc, scolex. [file 13071_2024_6222_MOESM1_ESM.pdf]

**(A)**

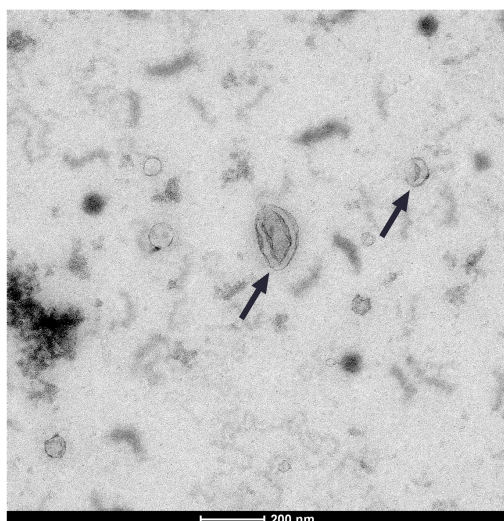

**(B)**

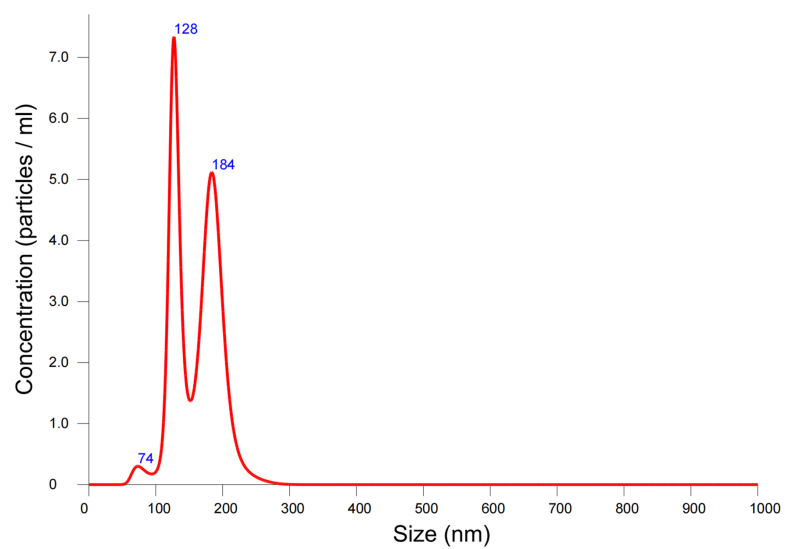

Supplement: Supplementary file 2 — Additional file 2: Figure S2. Transmission electron microscopy (A) and nanoparticle tracking analysis (B) of extracellular vesicles originating from HF. [file 13071_2024_6222_MOESM2_ESM.pdf]

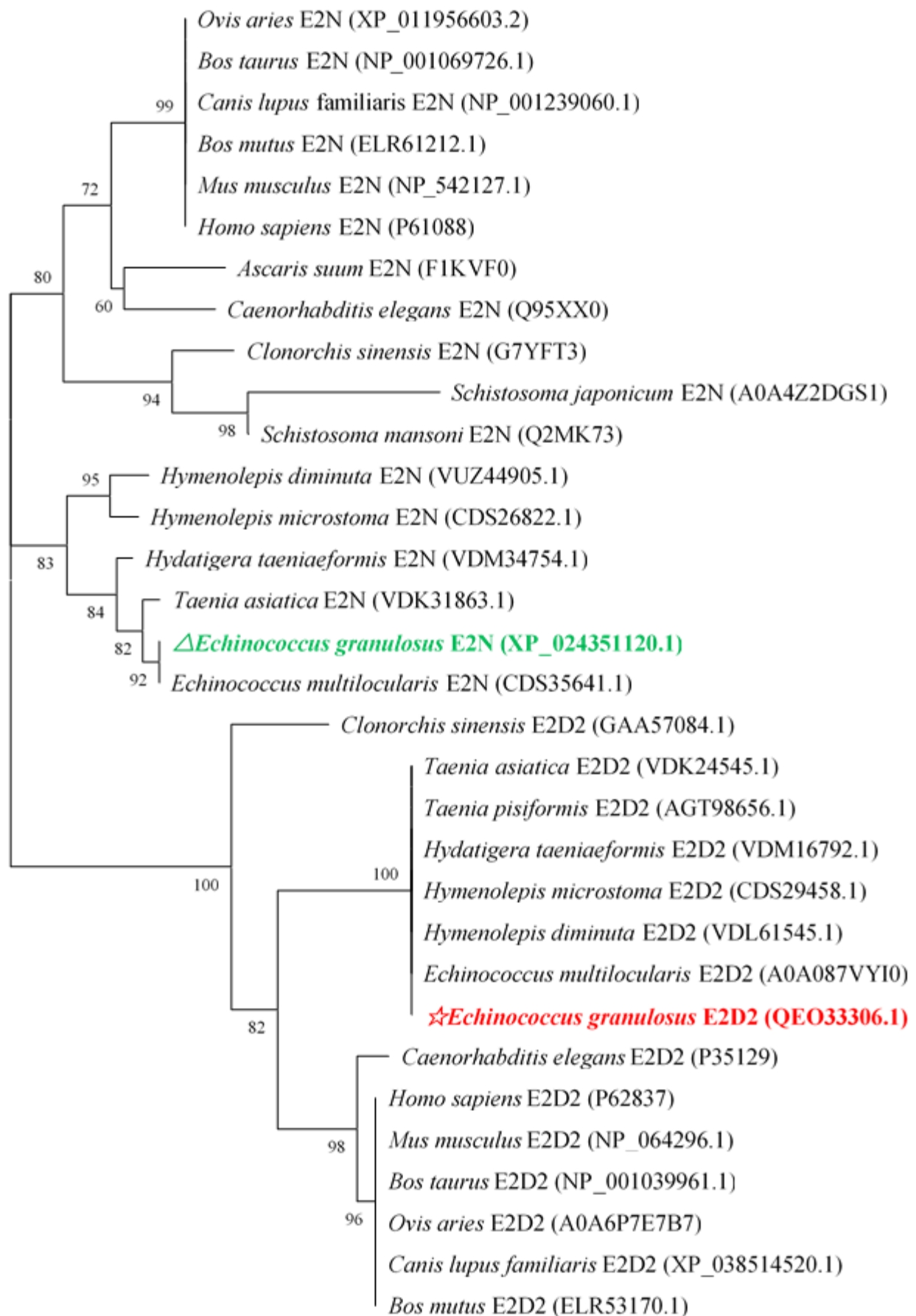

0.05

Supplement: Supplementary file 3 — Additional file 3: Figure S3. The phylogenetic tree (ML) of EgE2D2 and EgE2N. [file 13071_2024_6222_MOESM3_ESM.pdf]
